# Editorial: CaRe Me: improving the cardio-renal-metabolic care of patients through clinical and translational research

**DOI:** 10.3389/fmed.2024.1462223

**Published:** 2024-07-19

**Authors:** José Jesús Broseta, Elena Cuadrado-Payán, Diana Rodríguez-Espinosa, Pedro Ventura-Aguiar, Pedro Caravaca-Pérez

**Affiliations:** ^1^Department of Nephrology and Renal Transplantation, Hospital Clínic Barcelona, Barcelona, Spain; ^2^Department of Cardiology, Hospital Clínic Barcelona, Barcelona, Spain

**Keywords:** Cardiovascular-Renal-Metabolic Syndrome (CRMS), heart failure (HF), cardiovascular disease (CVD), chronic kidney disease (CKD), cardiometabolic, cardiorenal, diabetes, obesity

## 1 Introduction

The prevalence of chronic diseases is rising due to contemporary sedentary lifestyles, poor eating habits, and increased survival rates after cardiovascular events. Conditions such as heart failure (HF), type 2 diabetes mellitus, obesity, non-alcoholic fatty liver disease (NAFLD), and chronic kidney disease (CKD) pose significant health challenges in the 21st century. The economic burden of these conditions is growing, highlighting the urgent need for innovative management. Despite recent technological and medical advancements offering hope for improving patient outcomes and reducing healthcare costs (1–3), a deeper understanding of the underlying shared mechanisms and interconnectedness of these once-thought-of-as singular pathologies remains a critical and relevant area of scientific research in today's world.

## 2 Advances in the conceptualization of Cardiovascular-Renal-Metabolic Syndrome

The American Heart Association (AHA) has recently introduced the concept of Cardiovascular-Renal-Metabolic Syndrome (CRMS), providing a significant opportunity to improve multidisciplinary approaches and early intervention in the risk stratification, prevention, and treatment of this complex syndrome. This framework recognizes the interplay of cardiovascular, renal, and metabolic risk factors, exacerbated by cardiovascular diseases (including HF, atrial fibrillation, coronary artery disease, stroke, and peripheral artery disease), CKD, and type 2 diabetes, with excessive or dysfunctional adiposity as the underlying issue (4, 5). Three publications within this Research Topic establish a solid foundation for a conceptual and actionable framework by examining the interrelationship between obesity and glomerular hyperfiltration and hypertrophy and exploring the causal relationships between kidney function and cardiovascular diseases, as well as between metabolic syndrome and kidney disease. Adapting these new concepts to our reality is crucial, promoting stronger collaboration between primary care and the various involved specialties and establishing multidisciplinary units for optimal, patient-centered management.

## 3 Contributions from the Research Topic

This Research Topic aims to advance knowledge on the complex interplay between cardiovascular, renal, and metabolic systems. It encompasses practical reviews and cutting-edge research, focusing on enhancing patient care. It features a wide array of studies, from practical approaches to specific subtopics, systematic reviews, and original research spanning clinical, translational, and basic sciences.

Cai et al. conducted a study titled “*A mendelian randomization study revealing that metabolic syndrome is causally related to renal failure*” to explore the causal link between metabolic syndrome and renal failure using a Mendelian randomization approach. Their research, utilizing genetic databases, found a significant causal association between metabolic syndrome and renal failure, particularly with components like waist circumference and hypertension. These results underscore the importance of managing metabolic syndrome to prevent renal failure progression, especially in patients with hypertension or obesity.

In their study titled “*Kidney function and cardiovascular diseases: a large-scale observational and Mendelian randomization study*,” Hu et al. conducted a large-scale observational and Mendelian randomization study to investigate the causal relationship between kidney function and cardiovascular diseases. The study used data from the eICU Collaborative Research Database and found that while baseline cardiovascular diseases increased the risk of developing acute kidney injury during hospitalization, the reverse causal effect of kidney function on cardiovascular diseases was less definitive. The Mendelian randomization analysis indicated that genetically predicted atrial fibrillation and HF were linked to an increased risk of CKD and lower estimated glomerular filtration rate (eGFR). However, there was insufficient evidence to firmly establish causality from kidney function to cardiovascular diseases, underscoring the need for more mechanistic studies.

The study “*Association of high estimated glomerular filtration rate with risk of atrial fibrillation: a nationwide cohort study*” by Kang et al. analyzed the link between elevated eGFR and the risk of developing atrial fibrillation using a nationwide cohort study from Korea. The study analyzed data from over 2.6 million participants and found that both low (< 30 mL/min/1.73 m^2^) and high (>120 mL/min/1.73 m^2^) eGFR values were associated with an increased risk of atrial fibrillation. In contrast, participants in the 10th decile of eGFR (>113.41 mL/min/1.73 m^2^) showed a decreased risk. These findings indicate that while high eGFR levels are generally linked to a lower risk of atrial fibrillation, both extremes of eGFR could indicate varying cardiovascular risks, emphasizing hyperfiltration's relevance and the need for nuanced clinical assessment.

The article “*Glomerular hyperfiltration and hypertrophy: an evaluation of maximum values in pathological indicators to discriminate “diseased” from “normal”*” by Kataoka et al. examines glomerular hyperfiltration and hypertrophy roles in the progression of CKD, especially in obesity-related kidney pathology. The study emphasizes sustained glomerular hyperfiltration and hypertrophy lead to severe glomerular injury and kidney damage, highlighting pathological mechanisms, including adipose tissue inflammation, dyslipidemia, insulin resistance, chronic systemic inflammation, oxidative stress, and overactivation of the sympathetic nervous system and the renin-angiotensin-aldosterone system. The study aims to enhance the prediction and understanding of kidney failure by focusing on extreme values, such as maximal glomerular diameter. This underscores the potential usefulness of advanced glomerular size metrics and highlights the need for further research.

Finally, in “*Case report: Alternative approach for management of refractory volume overload in heart failure: usefulness of venous leg compression*,” Macián et al. detail a novel approach using venous leg compression for refractory volume overload in HF patients. They describe a patient with persistent peripheral edema despite high-dose diuretics. After applying venous leg compression, the patient significantly improved, resolving edema and losing weight. This case highlights the potential of venous leg compression as an effective complement to traditional diuretic therapy, particularly in cases resistant to standard treatments. The report emphasizes the need for further investigation into this technique's broader applicability in managing HF-related fluid overload.

## 4 Conclusion

The contributions in this Research Topic advance our understanding of the intricate relationships between cardiovascular, renal, and metabolic conditions. By integrating diverse research approaches and findings, these studies collectively enhance our knowledge of cardio-renal-metabolic syndrome, highlight the importance of multidisciplinary management strategies, and shed light on how shared mechanisms phenotypically differ in various situations.

To tackle these intertwined health issues, additional research is necessary to understand their cause-and-effect relationships and create comprehensive management plans. Research on new treatments, technologies, and patient-centered approaches, will be essential in advancing our knowledge and enhancing patient care.

## Author contributions

JB: Conceptualization, Data curation, Formal analysis, Funding acquisition, Investigation, Methodology, Project administration, Resources, Software, Supervision, Validation, Visualization, Writing – original draft, Writing – review & editing. EC-P: Conceptualization, Data curation, Formal analysis, Funding acquisition, Investigation, Methodology, Project administration, Resources, Software, Supervision, Validation, Visualization, Writing – original draft, Writing – review & editing. DR-E: Conceptualization, Data curation, Formal analysis, Funding acquisition, Investigation, Methodology, Project administration, Resources, Software, Supervision, Validation, Visualization, Writing – original draft, Writing – review & editing. PV-A: Conceptualization, Data curation, Formal analysis, Funding acquisition, Investigation, Methodology, Project administration, Resources, Software, Supervision, Validation, Visualization, Writing – original draft, Writing – review & editing. PC-P: Conceptualization, Data curation, Formal analysis, Funding acquisition, Investigation, Methodology, Project administration, Resources, Software, Supervision, Validation, Visualization, Writing – original draft, Writing – review & editing.

## References

[1] Vaduganathan M Mensah GA Turco JV Fuster V Roth GA. The global burden of cardiovascular diseases and risk: a compass for future health. J Am Coll Cardiol. (2022) 80:2361–2371. 10.1016/j.jacc.2022.11.005 36368511

[2] Chew NWS Ng CH Tan DJH Kong G Lin C Chin YH. The global burden of metabolic disease: data from 2000 to 2019. Cell Metab. (2023) 35:414–428.e3. 10.1016/j.cmet.2023.02.003 36889281

[3] Bello AK Okpechi IG Levin A Ye F Damster S Arruebo S. An update on the global disparities in kidney disease burden and care across world countries and regions. Lancet GlobHealth. (2024) 12:e382–e395. 10.1016/S2214-109X(23)00570-3 38365413

[4] Cases A Broseta JJ Marqués M Cigarrán S Julián JC Alcázar R. La definición del síndrome cardiovascular-reno-metabólico (cardiovascular-kidney-metabolicsyndrome) y su papel en la prevención, estatificación del riesgo y tratamiento. Una oportunidad para la Nefrología. Nefrologí*a*. (2024). 10.1016/J.NEFRO.2024.05.001 [Epub ahead of print].

[5] Ndumele CE Rangaswami J Chow SL Neeland IJ Tuttle KR Khan SS. Cardiovascular-kidney-metabolic health: a presidential advisory from the American Heart Association. Circulation. (2023) 148:1606–1635. 10.1161/CIR.0000000000001184 37807924

